# Nanostructured Electrode Materials for Electrochemical Capacitor Applications

**DOI:** 10.3390/nano5020906

**Published:** 2015-06-02

**Authors:** Hojin Choi, Hyeonseok Yoon

**Affiliations:** 1 Department of Polymer Engineering, Graduate School, Chonnam National University, 77 Yongbong-ro, Buk-gu, Gwangju 500-757, Korea; E-Mail: iyzvnzzang@gmail.com; 2 School of Polymer Science and Engineering, Chonnam National University, 77 Yongbong-ro, Buk-gu, Gwangju 500-757, Korea

**Keywords:** nanomaterials, electrochemical capacitors, electric double-layer capacitors (EDLCs), pseudocapacitors, hybrid capacitors

## Abstract

The advent of novel organic and inorganic nanomaterials in recent years, particularly nanostructured carbons, conducting polymers, and metal oxides, has enabled the fabrication of various energy devices with enhanced performance. In this paper, we review in detail different nanomaterials used in the fabrication of electrochemical capacitor electrodes and also give a brief overview of electric double-layer capacitors, pseudocapacitors, and hybrid capacitors. From a materials point of view, the latest trends in electrochemical capacitor research are also discussed through extensive analysis of the literature and by highlighting notable research examples (published mostly since 2013). Finally, a perspective on next-generation capacitor technology is also given, including the challenges that lie ahead.

## 1. Introduction

Electrochemical capacitors are a special class of electric energy storage devices that are based on nonfaradaic and/or faradaic charging/discharging at the interface between an electrode and an electrolyte [1,2]. In other words, they take advantage of ion adsorption and redox reactions to store electric energy in the electrode. They can complement or replace batteries used in electric energy storage devices and harvesting applications when high power delivery or uptake is required. The different properties of each type of electrochemical capacitor impart unique characteristics to such devices and enable their unique applications [3]. Two primary attributes of an electrochemical capacitor are its energy and power density, both of which are mostly expressed as a quantity per unit weight. Since the energy stored in electrochemical capacitors is related to the charge arising from the potential difference at each interface, especially in the case of nonfaradaic charge storage, they can offer more rapid charge/discharge rates as compared with batteries, whereas their energy density is lower than that of batteries [4]. Many researchers have made an effort to improve the energy density while maintaining high power density. Current research on electrochemical capacitors can be categorized into three device types: electric double-layer capacitors (EDLCs), pseudocapacitors, and hybrid capacitors [5].

In most cases, EDLCs are based on porous carbon electrode materials. EDLCs are often referred to as supercapacitors or ultracapacitors that electrostatically store the charge by using reversible adsorption of the electrolyte ions onto electrochemically stable, high-surface-area carbonaceous electrodes. The specific surface area of EDLCs is enlarged by making the bulk of the carbon material porous [6]. In principle, the main requirements for EDLC electrodes include: (i) fast charge/discharge rate; (ii) large potential window; (iii) high conductivity; and (iv) large effective surface area [7,8].

Pseudocapacitive materials show fast redox reactions during the charge/discharge process at their surface. The fact that charge storage is based on a redox process means that pseudocapacitors have some battery-like behavior (faradaic reaction) in their charge/discharge process [9,10]. Therefore, pseudocapacitors show higher capacitances than EDLCs, although they have somewhat slower charge/discharge rates than EDLCs. Pseudocapacitance is typically shown by materials such as conducting polymers and transition metal oxides. The interest is increasing in the development of improved materials for pseudocapacitors. To improve the capacitance of pseudocapacitors, four key factors are required: (i) doping of the conducting polymer to increase the redox state and conductivity; (ii) high charge/discharge rate; (iii) high surface area for the redox reaction; and (iv) a wide potential window [11,12].

Hybrid capacitors comprising EDLCs and pseudocapacitors combine their advantages, namely high energy and power densities [13,14]. The charge storage mechanisms in such devices are a combination of purely electrostatic adsorption–desorption phenomenon at the nonfaradaic electrode and a reversible faradaic reaction at the electrode. To achieve high energy density, hybrid capacitor systems comprising redox materials have been actively researched and developed in recent years [15]. Both EDLCs and pseudocapacitors are essential for fabricating high-performance hybrid capacitors. Electrochemical capacitors consist of electrolytes, separators, binders, and electrode materials. Here, we focus on the nanostructured electrode materials for use in the three different types of electrochemical capacitors, *i.e.*, EDLCs, pseudocapacitors, and hybrid capacitors. The latest important works and achievements in electrochemical capacitor research are highlighted to provide information on what material factors are critical in determining the performance of electrochemical capacitors. We will mainly cover studies that have been conducted over the last few years, with major emphasis placed on the literature from 2013 to the present day. Lastly, future trends in the research field will be discussed, along with remaining technical challenges.

## 2. EDLC Materials

Carbon materials are considered prospective electrode materials for industrialization. The advantages of carbon materials include large specific surface area, good electronic conductivity, and high chemical stability [16]. The charge storage mechanism of carbon-based electrode materials mostly conforms to that of EDLCs. The important factors influencing their electrochemical performance are specific surface area, pore-size distribution, pore shape and structure, electrical conductivity, and surface functionality. Among these, specific surface area and pore-size distribution are the two most important factors affecting the performance of EDLCs [17].

### 2.1. Porous Carbon

**Figure 1:**
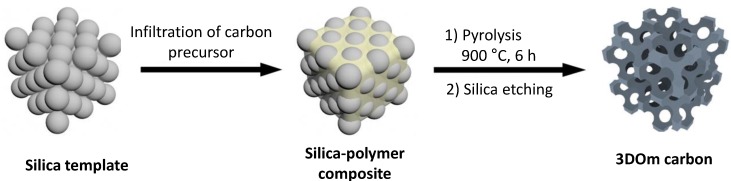
Schematic of fabrication of three-dimensionally ordered mesoporous (3DOm) carbon. With permission from [21]; Copyright 2013, American Chemical Society.

To realize the enhanced performance that stems from increasing the surface area of electrode materials, the pore size of the materials should be limited to the range of subnanometers to a few nanometers [18]. Extensive research has been conducted on developing porous structures [19]. The small pore sizes of carbon materials result in large specific surface areas. However, at a certain, optimal pore size, the effective surface area of the double layer is maximized while maintaining the optimal ion exchange with the electrolyte [20]. For example, a colloidal crystal templating method was optimized for the synthesis of three-dimensionally ordered mesoporous (3DOm) carbon with a well-defined geometry, a three-dimensional (3D) interconnected pore structure, and tunable pore sizes of 8–40 nm (Figure 1) [21]. To achieve precise control over the pore sizes in the carbon products, parameters were established for direct syntheses or seed growth of monodispersed silica nanospheres of specific sizes [22]. The 3DOms exhibited higher capacitance than conventional porous carbon electrodes. Moreover, using an ionic liquid as an electrolyte resulted in an increased cell voltage, which in turn enhanced the power density of the electrode [23].

These porous carbon materials have a synergistic effect when used in conjunction with other carbon materials [24]. A judicious combination of porous carbon and so-called nanocarbons, such as graphene [25], carbon nanotubes (CNTs) [26], and carbon nanofibers (CNFs) [27], could improve the capacitive performance based purely on the principle of an electrochemical double layer.

High-surface-area carbon nanotube (CNT)/microporous carbon composite materials were prepared for EDLC electrodes [28]. All-carbon-based CNT/microporous carbon core-shell nanocomposites have a high-surface-area microporous carbon shell. Therefore, these CNT/microporous carbon core-shell nanocomposites are promising electrode materials for EDLCs.

**Figure 2:**
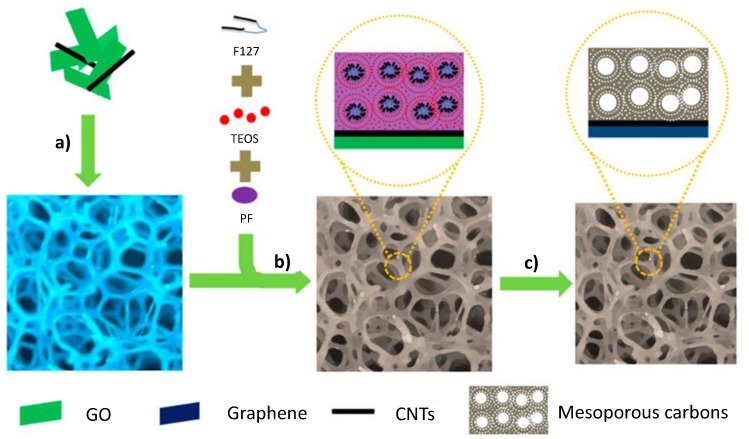
Fabrication of 3D hierarchically porous carbon–CNT–graphene ternary all-carbon foams (3D-HPCFs): (**a**) Impregnating sponge into the GOC solution; (**b**) Impregnating the resulting GOC/commercial sponge into a multicomponent ethanol solution of F127, phenol–formaldehyde resol, and tetraethoxysilane (TEOS) for self-assembly; (**c**) Carbonization and subsequent etching removal of SiO_2_ from TEOS. With permission from [29]; Copyright 2014, American Chemical Society.

In another case, porous carbon–CNT–graphene ternary all-carbon foams were obtained through multicomponent surface self-assembly of graphene oxide (GO)-dispersed pristine CNTs (GOCs) supported on a commercial sponge [29]. The GO acted as a “surfactant” that dispersed the CNTs, thereby preserving their excellent electronic structure and preventing the aggregation of graphene, which resulted in an overall improvement of the conductivity. The fabrication of 3D hierarchically porous carbon–CNT–graphene ternary all-carbon foams (3D-HPCFs) is illustrated in Figure 2. The large number of high-surface-area mesopores promotes ion transport/charge storage.

### 2.2. CNFs, CNTs, and Graphene

**Figure 3:**
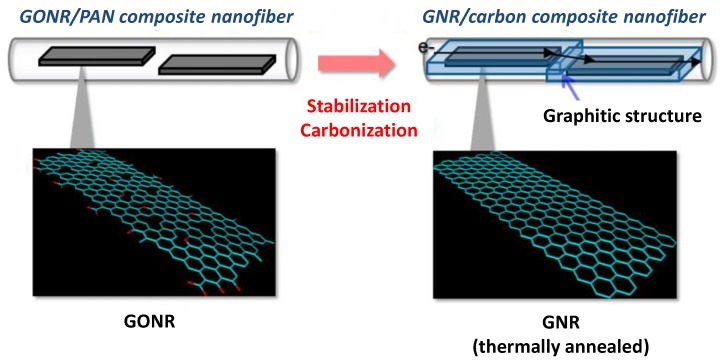
Schematic of the fabrication process of graphene nanoribbon (GNR)/carbon nanofiber (CNF) via carbonization. With permission from [33]; Copyright 2013, American Chemical Society.

Graphene has a maximum theoretical specific surface area of *ca.* 2600 m^2^·g^−1^, which is twice that of single-walled CNTs and substantially higher than those of most carbon black and activated carbons [30]. Graphene is a unique and attractive electrode material owing to its atom-thick two-dimensional (2D) structure and excellent properties. However, graphene sheets easily form irreversible agglomerates and restack to the graphitic structure. To overcome this problem, graphene can be hybridized with CNTs, CNFs, and porous carbon. Various carbon hybrids have been fabricated by electrospinning followed by heat treatment [31], since electrospinning can readily create multicomponent nanofibers as the carbon precursor [32]. Graphene nanoribbon (GNR)/carbon composite nanofibers have been prepared by electrospinning from polyacrylonitrile (PAN)-containing GO nanoribbons (GONRs) and successive twisting and carbonization (Figure 3) [33].

Many studies have shown that highly oriented CNFs have a large surface area and excellent electrical properties [34]. PAN-containing GONR nanocomposites also become highly oriented during electrospinning. In addition, GONRs have been converted to an all-carbon material, GNR/CNF, by carbonization [35]. Both the graphene ribbons and CNFs exhibit high performance as electrochemical capacitors; it is expected that the structural synergistic effect would further improve this performance. Various methods of fabricating these graphene fiber materials have been investigated. Graphene-coated nanotube aerogels, a type of graphene fiber material, have been developed by coating the nodes of an isotropic single-wall carbon nanotube network within an aerogel with a few layers of graphene. These graphene-based CNT and CNF materials are especially important for textile-enabled materials and devices. They can also act as the building blocks for forming 2D and 3D macroscopic structures for EDLCs [36,37]. The use of CNFs and CNTs has further enhanced the properties of microelectrochemical capacitors, thereby enabling the fabrication of flexible and adaptable devices [38].

**Figure 4:**
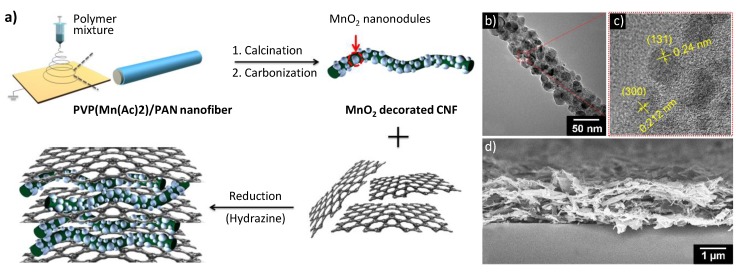
(**a**) Schematic of the fabrication of ternary hybrid nanostructures: MnO-decorated CNFs (MCNFs)/reduced graphene oxide (RGO) nanohybrids; (**b**) Typical transmission electron microscopy (TEM) image of MCNF; (**c**) High-resolution TEM image of an MCNF (lattice planes of γ-MnO_2_, such as (131) and (300), are marked); and (**d**) Cross-sectional scanning electron microscopy (SEM) image of MCNF/RGO nanohybrids (RGO 50 wt.%) deposited on a silicon wafer. With permission from [39]; Copyright 2013, Wiley-VCH Verlag GmbH & Co. KGaA.

Many researchers have reported on combinations of CNTs, CNFs, metals, and graphene. CNTs and CNFs are connected to the graphene layer through covalent bonds, leading to seamless, high-quality carbon material–graphene–metal interfaces [39]. Ternary hybrid nanostructures consisting of CNFs, manganese oxide (MnO), and graphene were recently fabricated, as shown in Figure 4. The metal oxide MnO is a pseudocapacitive element, which will be discussed in a subsequent section. MnO-decorated CNFs (MCNFs) were dispersed in an aqueous solution containing isolated GO sheets exfoliated from oxidized graphite. GO sheets are highly dispersible in aqueous solution owing to the oxygen-containing functional groups, and they have high affinity with CNFs because of their similar chemical structures. Thus, MCNFs were readily wrapped with GO sheets to yield a 3D nanohybrid architecture. Afterwards, GO was converted to reduced graphene oxide (RGO) by using hydrazine. Intercalated MCNFs improved the conductivity of the MCNF/RGO nanocomposite and facilitated ion diffusion by increasing the spacing between the graphene sheets. The RGO sheet is considered to act as a conductive channel in the nanohybrid. Additionally, the intercalation of CNFs between the RGO sheets induced a 3D opened geometry in the electrode, which allowed facile ion and charge transfer. Consequently, high-performance capacitive properties were observed for these new 3D structures [40,41,42,43].

**Figure 5:**
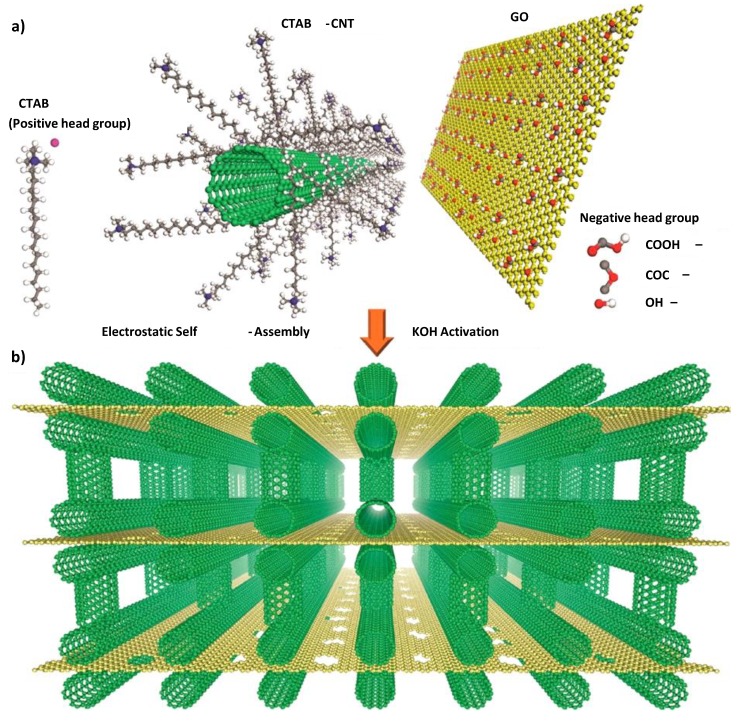
Schematic for fabricating the graphene/single-walled CNT hybrid nanostructure. (**a**) Cetyltrimethylammonium bromide (CTAB)-grafted CNTs are positively charged and the GO layers are negatively charged owing to their respective functional groups; (**b**) Schematic of the 3D CNT-bridged graphene block. KOH activation generates nanoscale pores in the graphene layers, which are expected to provide a simple means of ion diffusion. With permission from [44]; Copyright 2015, American Chemical Society.

CNT-bridged graphene 3D building blocks were synthesized via the coulombic interaction between positively charged CNTs grafted by cationic surfactants and negatively charged GO sheets (Figure 5) [44]. The CNTs were intercalated into the nanoporous graphene layers to build pillared 3D structures, which increased the accessible surface area and allowed fast ion diffusion. Because of this unique 3D porous structure, the electrodes showed remarkable electrochemical performance in ionic liquid electrolytes [45].

**Figure 6:**
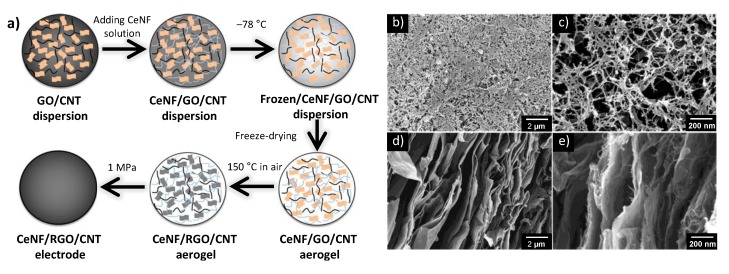
(**a**) Schematic of the fabrication process of a CeNF/RGO/CNT aerogel electrode. SEM images of compressed CeNF/RGO/CNT hybrid aerogel films, (**b** and **c**) bottom surface of the aerogel film, and (**d** and **e**) cross section of the aerogel film. With permission from [46]; Copyright 2015, American Chemical Society.

However, it is difficult to prepare electrodes by using uniform nanostructured CNTs on special substrates, especially those with porous surface structures. Cellulose is the most abundant and sustainable natural polymer. Cellulose nanofibrils (CeNFs) derived from cellulose have high aspect ratios, excellent mechanical properties, excellent flexibility, and superior hydrophilicity [46]. A recent work reported the fabrication of a unique CeNF/carbon nanohybrid aerogel electrode material, shown in Figure 6. The CeNF-based aerogel possessed a porous structure and an extremely high porosity (resulting in ultralow density and a high specific surface area), as well as excellent electrolyte-absorption properties. Furthermore, the hydrophilicity of the CeNFs in the aerogel improved the contact between the electrodes and electrolytes, and it provided diffusion channels for the electrolyte ions, thus enhancing the performance of the EDLCs.

Various polymer precursors have been used to make new types of carbon nanomaterials. The main characteristics of the resultant carbon nanomaterials depend on the polymer precursors. It is relatively easy to control the morphology and composition of polymers, which offers opportunities for producing carbon nanomaterials with controlled structures and properties [47,48]. Several researchers have fabricated electrode materials via heat treatment of GO/polymer hybrid precursors, such as GO/polypyrrole (PPy) nanowires, GO/PPy nanotubes, and GO/polyaniline (PANI). Heat treatment of the GO/polymer hybrids resulted in the formation of graphene-embedded all-carbon nanostructures. These carbonized nanohybrids exhibited good performance as EDLC electrode materials.

### 2.3. Summary

EDLCs can be applied in the case of stationary and mobile systems requiring high power pulses. Moreover, owing to their low time constant, they can quickly harvest energy, such as during deceleration or braking of vehicles. Although EDLCs are able to provide higher power with a longer cycle life, they suffer from a relatively low energy density. Therefore, the current research is mainly concerned with the optimization of the existing electrode materials and the development of new materials to improve energy density. Many different carbon forms, such as CNTs, CNFs, activated carbon powders, and carbonized materials, can be used as active materials in EDLC electrodes [49,50]. Table 1 summarizes the EDLC electrode materials developed recently and their electrochemical performance.

**Table 1:** EDLC materials and electrochemical performance.

Materials	Electrode System ^(1,2)^	Electrolyte	Current Density or Scan Rate	Potential Range (V)	Specific Capacitance (F·g^−1^)	Ref.
Carbon nanosheets	Two	^(3)^ EMIMBF_4_	1–10 A·g^−1^	3.5	155–242	6
Carbon nanosheets	Two	PVA–H_3_PO_4_ gel	0.25–1.3 A·g^−1^	0.8	4.9–29.2	7
*N*-doped carbon nanosheets	Two	1 M NaOH	0.75–7 A·g^−1^	1.0	150–180	8
Porous carbon nanowhiskers	Two	6 M KOH	0.5–30 A·g^−1^	1.0	125–210	16
*N*-doped nanoporous carbon	Three	6 M KOH	0.5–40 A·g^−1^	1.0	90–240	17
Nanofibers/mesoporous carbon	Three	0.5 M K_2_SO_4_	0.5–6 A·g^−1^	0.8	72–99	18
*N*-doped carbon nanospheres	Three	6 M KOH	0.5–40 A·g^−1^	1.0	62–194	19
Nanoporous carbon	Two	^(4)^ EMI–TFSI	0.5–25 A·g^−1^	3.5	80–178	21
*N*-doped nanoporous carbon	Three	6 M KOH	1–10 A·g^−1^	1.0	175–250	22
Mesoporous carbon nanosheets	Two	^(5)^ 1 M TEABF_4_/AN	0.1–120 A·g^−1^	2.7	100–130	24
GO-activate carbon	Two	EMIMBF_4_	1–10 A·g^−1^	3.5	110–135	25
Porous carbon/CNTs	Two	3 M H_2_SO_4_	0.1–50 A·g^−1^	0.9	125–237	26
Porous CNFs	Two	EMI-TFSI	5–100 mV·s^−1^	2.0	65–150	27
Porous CNTs	Three	1 M H_2_SO_4_	1–10 A·g^−1^	0.7	454–710	28
Nanoporous carbon foams	Two	6 M KOH	0.2–20 A·g^−1^	1.1	125–379	29
Carbonization of carbon hydrates	Two	5 M KOH	1 A·g^−1^	0.8	140	32
Carbonized hollow nanocarbon	Two	1 M KOH	1–20 A·g^−1^	0.9	160–183	35
Graphene-coated CNTs	Two	EMI-TFSI	0.01–10 A·g^−1^	3.0	60–130	36
Graphene/CNF	Three	1 M H_2_SO_4_	0.2 A·g^−1^	1.5	174	39
3D porous graphene-like carbon	Two	^(6)^ TEMABF_4_/PC	1–32 A·g^−1^	2.5	156–178	40
Mesoporous graphene nanoballs	Three	1 M H_2_SO_4_	5–100 mV·s^−1^	0.8	206	41
Graphene/CNT composite fibers	Two	0.5 M H_2_SO_4_	0.2–2 A·g^−1^	0.8	6–35	42
*N*-doped 3D nanoporous carbon	Two	0.5 M Na_2_SO_4_	0.5–20 A·g^−1^	1.0	226–304	43
Graphene/CNT	Two	EMIMBF_4_	10 A·g^−11^	4.0	199	44
Graphene/carbon	Two	EMIMBF_4_	1–10 A·g^−1^	3.5	160–190	45
RGO/CNT	Two	PVA–H_2_SO_4_ gel	0.5–4 A·g^−1^	1.0	180–252	46
Carbonized PPy nanostructures	Three	1 M H_2_SO_4_	5 mV·s^−1^	0.9	264	47
Carbonized PPy-CNTs	Three	1 M KCL	5–100 mV·s^−1^	1.5	40–140	48
Halogen-containing nanoporous carbon	Three	6 M KOH	0.5–40 A·g^−1^	1.0	110–313	49
Oxygen-rich nanoporous carbon	Two	1 M H_2_SO_4_	0.5–10 A·g^−1^	1.0	210–297	50

^(1)^ The “Two” indicates capacitor cells composed of two electrodes (anode/cathode), separator, and electrolyte, containing symmetric or asymmetric electrode configurations; ^(2)^ The “Three” indicates electrode measuring systems composed of working electrode, counter electrode, and reference electrode in an electrolyte; ^(3)^ EMIMBF_4_: 1-ethyl-3-methylimidazolium tetrafluoroborate; ^(4)^ EMI-TFSI: 1-ethyl-3-methylimidazolium bis(trifluoromethylsulfonyl)imide; ^(5)^ TEABF_4_/AN: tetraethylammonium tetrafluoroborate/acetonitrile; ^(6)^ TEMABF_4_/PC: triethylmethylammonium tetrafluoroborate/propylene carbonate.

## 3. Pseudocapacitive Materials

Various methods of preparing pseudocapacitive materials have been recently described [51,52]. Pseudocapacitive materials show redox reactions during the charge/discharge process, thereby resulting in pseudocapacitors whose capacitance is better than that of EDLCs [53,54,55]. In most cases, however, faradaic redox reactions for pseudocapacitance are confined to the surfaces of the electrode materials. Thus, rational nanostructuring of pseudocapacitive materials for enlarging the effective surface area is essential to obtaining enhanced pseudocapacitance [56].

### 3.1. Conducting Polymers

Precise control over the size and morphology of conducting polymers on the nanoscale is essential to improving the performance of pseudocapacitors. However, the highly unstable nature of polymers on this scale has hindered the development of polymer nanoarchitectures [57]. Nevertheless, numerous efforts have been made to fabricate polymer nanomaterials with well-defined sizes and morphologies, and various types of conducting polymer nanostructures have been fabricated in a controlled manner [10,58].

**Figure 7:**
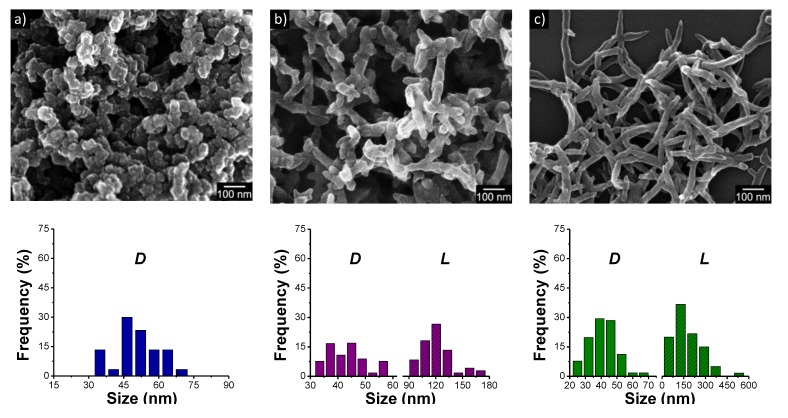
SEM images of polyaniline (PANI) nanostructures with different aspect ratios synthesized under the same stirring condition (200 rpm) and histograms showing their size distribution (D, diameter; L, length): (**a**) Nanospheres; (**b**) Nanorods; and (**c**) Nanofibers. With permission from [61]; Copyright 2012, American Chemical Society.

Owing to their unique properties and small dimensions, conducting polymer nanostructures have wide technical applications [59,60]. For example, Figure 7 shows the SEM images of three PANI nanostructures synthesized with different shapes. PANI has been extensively used as an electrode-active material in electrochemical capacitors. The capacitances of these PANI nanostructures were determined via charge/discharge cycling. For the same range of potentials, the discharging time increased in the order of nanospheres < nanorods < nanofibers, indicating that the nanofiber electrode has the highest specific discharge capacitance [61]. In other words, the capacitance of PANI nanostructures depends on their aspect ratio. The nanofibers also had faster electrode kinetics by virtue of the higher oxidation level and crystallinity. Lastly, interparticle resistance varied as a function of the aspect ratio of the nanostructures, which has been identified as one of the significant factors affecting the electrode performance. Several papers have similarly described the morphology effect of nanoparticles on the performance of pseudocapacitors [10,58,61]. As a result, judicious control of the morphology of nanostructures for electrode materials offers many possibilities of tailoring the performance of electrochemical capacitors.

Precise control of the nanostructure morphology is crucial to realizing a variety of next-generation technologies. Poly(3,4-ethylenedioxythiophene) (PEDOT) and multidimensional PPy nanotubes are only two examples of these promising nanostructures [62]. The surface of the former was decorated with substructures such as nanonodules and nanorods and the latter were fabricated as chemiresistors on a sacrificial nanofiber template by vapor deposition polymerization (VDP) [63]. These unique multidimensional nanostructures have enhanced surface-to-volume ratios, and their unique morphology and anisotropic geometry results in a high surface area and excellent charge-transport properties. These advantages are expected to induce a synergetic effect that enhances device performance, particularly in pseudocapacitors.

**Figure 8:**
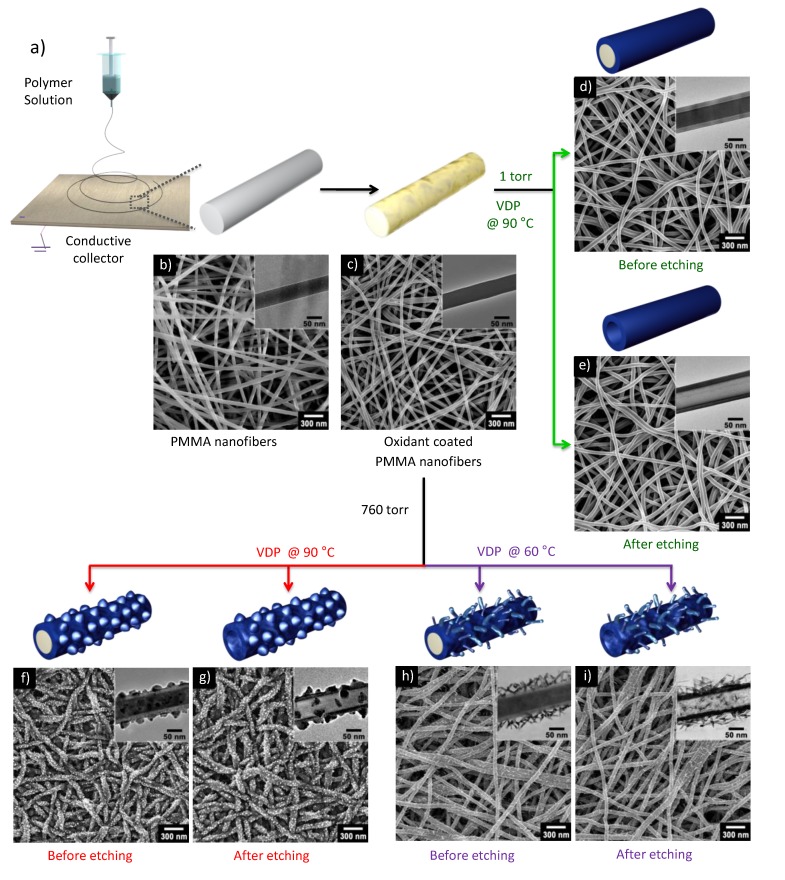
Multidimensional poly(3,4-ethylenedioxythiophene) (PEDOT) nanostructures with unique surface substructures. (**a**) Schematic of the synthetic routes for fabricating multidimensional PEDOT nanostructures; (**b**–**i**) The poly(methyl methacrylate) (PMMA) nanofibers serve as a template and substrate for the growth of PEDOT under different synthetic conditions (temperature and pressure). The morphologies of the resulting nanomaterials were characterized by SEM and TEM (right top inset images): PMMA nanofibers (**b**) before and (**c**) after ferric ion adsorption; PMMA/PEDOT nanofibers with a smooth layer surface (**d**) before and (**e**) after core etching; PMMA/PEDOT nanofibers with nanorod surface (**f**) before and (**g**) after core etching; PMMA/ PEDOT nanofibers with NN surface (**h**) before and (**i**) after core etching. With permission from [64]; Copyright 2012, American Chemical Society.

Electrospinning is a simple and scalable technique that can create continuous and aligned polymer nanofibers and microfibers under a large electric field. Electrospun nanofibers are composed of various polymer melts and solutions, which can serve as templates that direct the formation of conducting polymer nanofibers and nanotubes. In a recent study, ultrathin poly(methyl methacrylate) (PMMA) nanofibers with an average diameter of 60 nm were obtained through electrospinning for use as a template (Figure 8b); these nanofibers were then immersed in a ferric chloride solution in order to adsorb ferric ions onto the PMMA nanofibers (Figure 8c) [64]. Subsequent coating of PEDOT onto the PMMA nanofibers via VDP at controlled temperatures and pressures yielded core–shell-structured nanofibers (Figure 8d,f,h). Unique surface substructures such as nanonodules and nanorods were grown on the nanofibers by adjusting the major kinetic factors. Moreover, the PMMA was removed by selective solvent etching, which resulted in nanotubular structures (Figure 8e,g,i). These multidimensional nanofibers and nanotubes with surface substructures offer significant advantages when used as pseudocapacitors.

**Figure 9:**
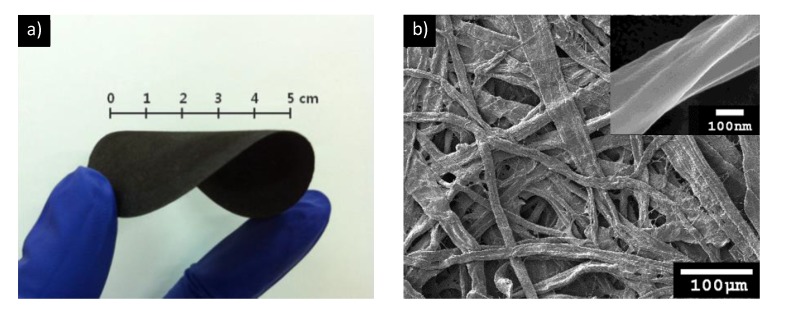
(**a**) Photograph showing the flexibility of a large-area, free-standing PPy/cellulose (PPCL) paper; (**b**) SEM images of a PPCL paper (inset: high-magnification image). With permission from [65]; Copyright 2014, The Royal Society of Chemistry.

VDP has attracted significant attention owing to its simplicity and controllability as compared to other techniques used to obtain nanostructured conducting polymers [65]. As a conjugated polymer, PPy shows practical potential for a diverse and promising range of future technologies. Free-standing, flexible, and large-area PPy/cellulose (PPCL) papers were readily prepared using VDP (Figure 9), and PPy coatings on cellulose microfibers were influenced by the properties of co-vapors introduced together with pyrrole during polymerization [66].

It is known that PPy stores electrical charges through a pseudocapacitive charge storage mechanism mediated by a redox reaction. Pseudocapacitance stems from reversible surface or near-surface reactions for charge storage. As a result, use of co-vapors in VDP has offered the possibility of tuning the physical properties of the deposited polymers, as well as the performance of the polymer-based electrochemical capacitors.

Conducting polymers can also be coated on metal nanoparticles to improve the electrochemical performance. Uniform coating of the metal nanoparticles with conducting polymer layers prevents interparticle aggregation and imparts unprecedented electrical, optical, and chemical properties to the nanoparticles.

**Figure 10:**
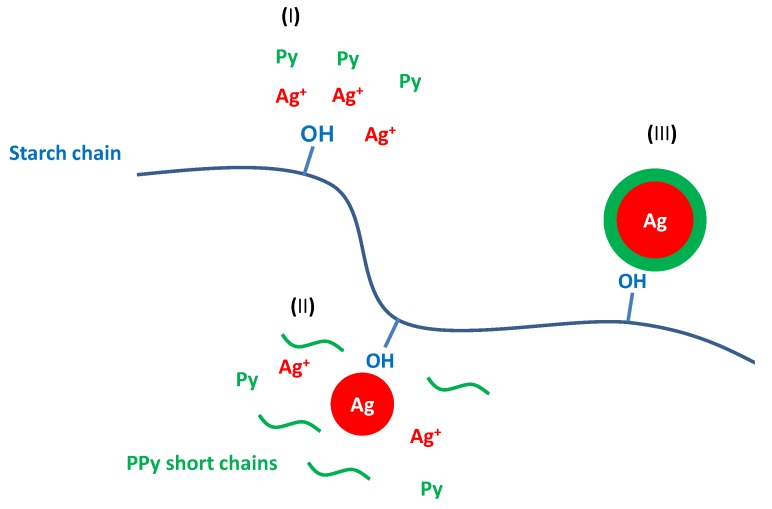
Schematic of the formation mechanism of Ag–PPy nanoparticles: the reaction process consisted of three stages (I, II, and III). With permission from [67]; Copyright 2012, American Chemical Society.

Ag–PPy core–shell nanoparticles were obtained in the form of a stable colloidal suspension through a simple one-pot synthesis [67]. Briefly, the silver nanoseed surfaces became the active sites for oxidation of the surrounding pyrrole monomers and PPy short chains, thereby resulting in the formation of a PPy shell (Figure 10). These bottom-up approaches offer an efficient and simple route for the fabrication of nanostructured metal/conducting polymer complexes.

### 3.2. Metal Oxides

Metal oxides are attractive pseudocapacitive electrode materials because of their easy processing [5]. Ruthenium oxides and manganese oxides have been widely studied for electrochemical capacitor applications [68,69]. However, the high cost of ruthenium oxides excludes them from wide practical application. Additionally, manganese oxides have several different crystalline structures, such as α-, β-, γ-, δ-, and ξ-MnO_2_, and unfortunately their capacitances highly depend on the crystalline structure [70,71,72,73,74]. Therefore, recent research trends have focused on exploring other transition metal oxides. For example, a binary transition metal sulfide, NiCo_2_S_4_, with a hollow hexagonal nanoplate structure was successfully prepared through a hydrothermal route with the aid of a sacrificial template. The NiCo_2_S_4_ gave a maximum specific capacitance of 437 F·g^−1^ in a KOH electrolyte at a current density of 1 A·g^−1^ [75]. However, poor conductivity of metal oxides poses a serious problem to the efficient use of these oxides in electrochemical capacitors. A nanocomposite composed of a metal oxide and a conductive material can be designed in order to overcome this drawback [66,76,77]. For example, it was found that CoO-based PPy nanowire electrodes had very high capacitance and good rate capability [78]. Using nanoporous silica as a template enables the fabrication of pseudocapacitor electrodes, which have narrow particle size and pore-size distributions, large surface area, and large pore volume. As compared to normal PPy, enhanced conductivity and surface area were observed due to the synergistic effect of the nanohybrid structures. As a result, the CoO/PPy nanowires showed excellent pseudocapacitance behavior [79].

**Figure 11:**
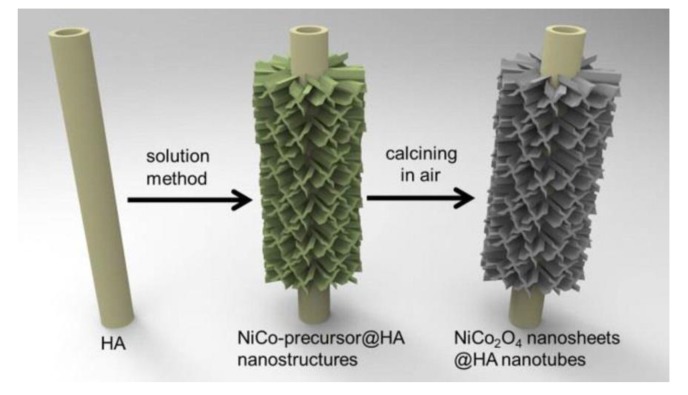
Schematic of the synthetic procedure of NiCo_2_O_4_ nanosheets@halloysite nanotube hybrid nanostructures. With permission from [84]; Copyright 2012, American Chemical Society.

Extensive research has also focused on maximizing the specific surface area of metal oxides through hierarchical structuring of the composite [80,81,82,83]. As an example, Ding and Yang reported the preparation of a 1D hierarchical nanostructure of NiCo_2_O_4_ nanosheets@halloysite nanotubes through simple co-precipitation followed by thermal annealing (Figure 11) [84]. The hierarchical nanostructure had an excellent specific capacitance of 1728 F·g^−1^ even after 8600 cycles at a current density of 10 A·g^−1^.

### 3.3. Summary

In recent years, various methods for fabricating pseudocapacitive materials have been reported. Table 2 summarizes the pseudocapacitive electrode materials and their electrochemical performance. Owing to the redox reaction-mediated charge storage, pseudocapacitive materials have higher energy density than their EDLC counterparts. The enhanced surface area and redox activity was achieved primarily by controlling the morphology and by combining materials. However, pseudocapacitive materials, especially conducting polymers, undergo severe volumetric changes during charge/discharge processes, often leading to structural deterioration and rapid decay of the capacitance. Long-term cycling stability is, therefore, a barrier to the wide use of pseudocapacitors in practical applications.

**Table 2:** Pseudocapacitor materials and electrochemical performance.

Materials	Electrode System ^(1,2)^	Electrolyte	Current Density or Scan Rate	Potential Range (V)	Specific Capacitance (F·g^−1^)	Ref.
CoCl_2_ nanostructures	Three	2 M KOH	1 A·g^−1^	0.45	1962	9
PANI nanotubes	Two	1 M H_2_SO_4_	1–30 A·g^−1^	0.5	477–896	10
PPy-clay core–shell nanoarrays	Three	1 M KOH	1–20 A·g^−1^	1.2	1750–2342	11
Polythiophene nanostructures	Two	0.5 M TEABF_4_	40–100 mV·s^−1^	4.0	75–250	12
NiO nanoblocks	Three	1 M KOH	1.11–111 A·g^−1^	0.6	680–1336	51
PPy–sepiolite nanocomposites	Three	1 M KCl	3 mA·cm^−2^	1.0	165	52
ZnCo_2_O_4_ nanorods/Ni foams	Two	PVA-KOH gel	1–20 A·g^−1^	1.0	1015–1400	53
NiCo_2_(OH)_6_ nanotubes	Two	1.9 M KCL 0.1M KOH	10–100 A·g^−1^	0.5	169–200	54
3D Co_3_O_4_ nanonetworks	Three	6 M KOH	2–100 mV·s^−1^	1.5	546–1049	55
Ni_2_(CO_3_)(OH)_2_ nanosheets	Three	3 M KOH	0.5–10 A·g^−1^	0.4	612–1178	56
PPy–PANI double-wall nanotubes	Three	1 M H_2_SO_4_	5–250 mV·s^−1^	0.6	366–693	58
PANI nanofibers	Three	1 M H_2_SO_4_	0.1–10 A·g^−1^	0.8	20–192	61
PPy nanofibrils	Three	1 M H_2_SO_4_	0.1 A·g^−1^	0.7	280	62
Hollow NiCo_2_S_4_ nanoplates	Three	3 M KOH	1–20 A·g^−1^	0.5	231–437	63
α-Fe_2_O_3_/MnO_2_ nanowires	Three	0.7 M H_3_BO_3_	1–50 A·g^−1^	0.6	480–838	70
2D TiS_2_ nanocrystals	Three	1 M LiClO_4_	0.5–10 A·g^−1^	1.2	320–470	71
CoAl/PEDOT nanoarrays	Three	6 M KOH	1–40 A·g^−1^	0.6	424–672	76
Au-MnO_2_ core–shell nanomesh	Two	PVA-LiClO_4_ gel	0.56 A·g^−1^	2.0	524	77
CoO/PPy nanowires	Two	3 M NaOH	1–50 mA·cm^−2^	1.6	647–2223	78
V_2_O_5_-PPy nanofibers	Two	PVA-LiCl gel	4.5 mA·cm^−2^	2.0	412	79
CuO nanowires	Three	2 M KOH	1–5 A·g^−1^	0.45	102–118	80
Nanoporous Ni(OH)_2_ films	Two	6 M KOH	0.9–50 A·g^−1^	1.6	20–192	81
β-Co(OH)_2_ nanosheets	Two	2 M KOH	1–25 A·g^−1^	0.5	1530–2080	82
Co_3_O_4_ nanostructures	Two	2 M KOH	0.5–2.5 A·g^−1^	0.8	150–476	83
NiCo_2_O_4_ nanosheets	Three	2 M KOH	6–30 A·g^−1^	0.5	1500–1886	84

^(1)^ The “Two” indicates capacitor cells composed of two electrodes (anode/cathode), separator, and electrolyte, containing symmetric or asymmetric electrode configurations. ^(2)^ The “Three” indicates electrode measuring systems composed of working electrode, counter electrode, and reference electrode in an electrolyte.

## 4. Hybrid Capacitive Materials

Carbon materials have excellent electrical properties such as conductivity, power density, and long-term cycling stability; however, overcoming their low energy density has been the subject of intensive research [85,86]. The specific capacitance of carbon materials can be improved by mixing them with pseudocapacitive active materials [87]. The pseudocapacitance of electronically conducting polymers stems from rapid and reversible oxidation and reduction processes. EDLCs composed of pseudocapacitive-based nanostructures can store charge on the electrode surface both through a double layer and via a redox reaction. The low cycling stability of pseudocapacitive materials can be overcome through the use of carbon materials. Several active materials, including conducting polymers PPy, PANI, or metal oxides, have been directly grown on carbon owing to the properties imparted by these carbon-based materials [88,89,90]. These properties could be combined with those of the transition metal oxides and polymers, leading to the development of a new brand of electrochemical capacitors.

### 4.1. Coupling EDLC and Pseudocapacitive Materials

Carbon-coated conducting polymers have, in general, excellent cycling stability, as well as high energy and power density [91]. More importantly, carbon materials enhance the corresponding cycling stability and prevent the structural breakdown of pseudocapacitive conducting polymers [92]. The energy density is also improved with the aid of the pseudocapacitance stemming from the conducting polymers.

For example, exceptional capacitance retention of 95% and 85% after 10,000 cycles were recently reported for carbon-coated PANI and PPy [93]. These retention rates are the best values ever obtained for polymer-based pseudocapacitive electrodes in an aqueous electrolyte. These results show that the carbon material was very effective in maintaining the structures of the PANI and PPy nanowire electrodes during charge/discharge cycling, thereby resulting in excellent capacitance retention rates. The carbon-coated PANI and PPy electrodes exhibited similar pseudocapacitive behavior and specific capacitance as compared to bare polymer samples. Furthermore, the excellent electrochemical performance of the polymer electrodes was maintained, even with improvement in cycling stability [94].

**Figure 12:**
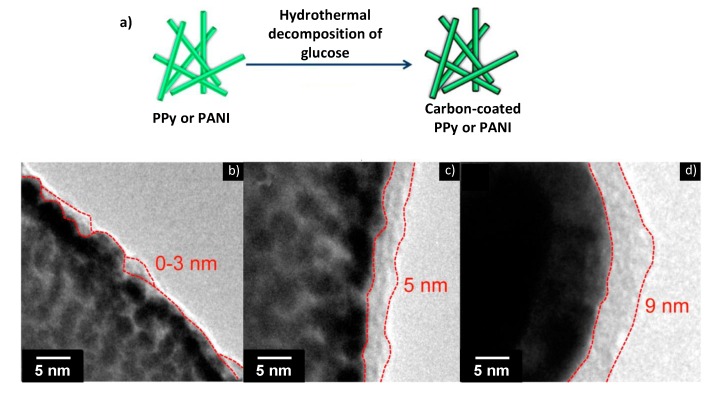
(**a**) Schematic of the carbonaceous coating procedure using glucose as the carbon precursor. (**b**–**d**) TEM images of carbon-coated PPy samples with different carbonaceous shell thicknesses, which was controlled via the reaction time for the hydrothermal deposition of glucose: (**b**) 1, (**c**) 2, and (**d**) 3 h. With permission from [93]; Copyright 2014, American Chemical Society.

Nickel- and manganese-based oxides/hydroxides have high theoretical specific capacitance. They are also inexpensive, naturally abundant, and environmentally friendly. Metal–oxide and graphene–oxide hybrid capacitors have many outstanding properties. Nickel hydroxide-manganese dioxide-RGO [Ni(OH)_2_–MnO_2_–RGO] ternary hybrid spherical powders were fabricated as electrode materials [95]. These materials have a highly porous nanostructure, relatively high specific surface area, and well-defined spherical morphology. In addition, the synergetic effect of Ni(OH)_2_, MnO_2_, and RGO resulted in significantly enhanced specific capacitance of the electrode materials composed of these novel Ni(OH)_2_–MnO_2_–RGO ternary hybrid spheres

### 4.2. Asymmetric Hybrid Capacitors

The development of asymmetric capacitors for improving the energy and power density of electrochemical capacitors has been extensively studied. Combining the advantages of long-term cycling, a fast and reversible nonfaradaic negative electrode (−), and a high-capacitive positive faradaic electrode (+), asymmetric capacitors should fulfill the requirements of high energy and power density [96,97,98].

**Figure 13:**
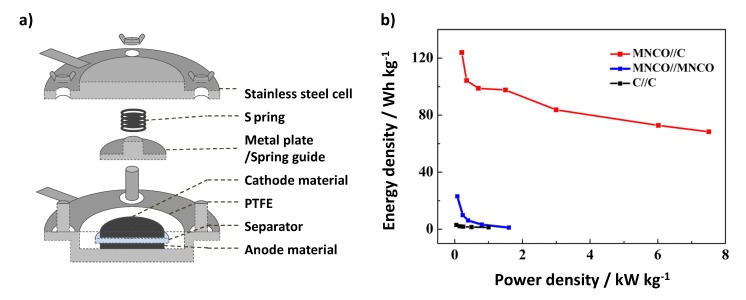
(**a**) Schematic of a representative asymmetric cell. With permission from [100]; Copyright 2014, The Royal Society of Chemistry. (**b**) Ragone plots of the as-fabricated asymmetric MNCO//carbon black, symmetric MNCO//MNCO, and symmetric carbon black//carbon black electrochemical capacitors. With permission from [99]; Copyright 2012, American Chemical Society.

To fabricate asymmetric capacitors, Mn–Ni–Co ternary oxide (MNCO) nanowires were synthesized by a simple hydrothermal method [99]. The exceptional performance of these capacitors was a result of their nanowire architecture, which provided a large reaction surface area and fast ion and electron transfer. An asymmetric hybrid capacitor with high energy density was assembled successfully by employing an MNCO nanowire (+)//carbon black (−) cell. Figure 13 shows an image of an asymmetric hybrid capacitor and the improved electrochemical performance of an asymmetric cell.

**Figure 14:**
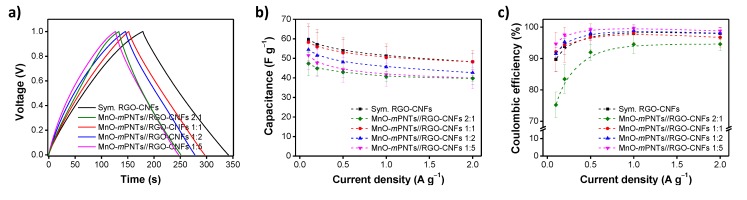
Effect of electrode weight: (**a**) Representative charge/discharge curves measured at 0.1 A·g^−1^; (**b**) Specific capacitances; and (**c**) Coulombic efficiencies measured at different current densities. With permission from [100]; Copyright 2014, The Royal Society of Chemistry.

In another study, MnO_2_–PEDOT nanotubes (mPNTs) were tested in asymmetric hybrid cell configurations, without using binders or conductive fillers, where RGO–CNFs were employed as an electric double-layer electrode material [100]. Surface redox reactions between the transition metal oxides and PEDOT resulted in the desired pseudocapacitance. Moreover, PEDOT has a nano-scale hierarchical structure, which provides a large effective surface area and efficient charge transfer. CNFs were intercalated between the RGO sheets to prevent the restacking of the sheets and to increase the nonfaradaic charge storage. The asymmetric MnO_2_–mPNTs (+)//RGO–CNFs (−) cell also exhibited superior specific capacitance, cycling stability, and coulombic efficiency as compared to symmetric cells comprising the MnO_2_–mPNT/RGO–CNF combination. Furthermore, the capacitive performance of the asymmetric MnO_2_–mPNT (+)//RGO–CNF (−) cells were examined in terms of the weight ratio of the positive/negative electrode materials. As Figure 14 shows, the capacitance of the asymmetric MnO_2_–mPNT//RGO–CNF cell decreased and the coulombic efficiency increased with increasing weight of the RGO–CNFs. The capacitive performance of the asymmetric cells was, therefore, highly dependent on the weight ratio of the electrode [101,102].

### 4.3. 3D Nanostructured Graphene-Based Capacitors

**Figure 15:**
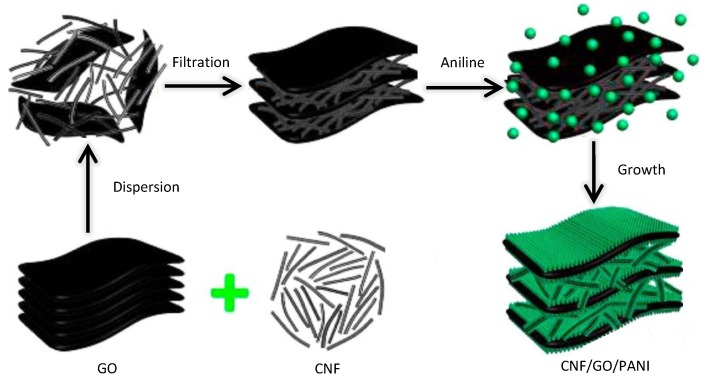
Formation mechanism of the CNF/GO/PANI film. With permission from [103]; Copyright 2013, American Chemical Society.

3D nanostructured hybrid materials, with better interfacial contact and volume utilization, have stimulated the development of several energy-efficient technologies via *in situ* polymerization of aniline onto a porous CNF/GO template (Figure 15). Such materials can be used for the facile fabrication of 3D PANI-modified CNF/GO hybrid electrodes, with the template imparting excellent conductivity and flexibility to the electrodes [103]. Specifically, CNFs significantly reduce the aggregation degree of GO, thereby forming a porous structure, which in turn substantially improves the utilization surface of GO. The *in situ* facial deposition process results in 3D hierarchically nanostructured PANI/CNF/GO composite electrodes with unique acanthine-style PANI nanowires covering the CNF/GO supports. PPy and other conducting polymers and metal oxides can also be used in hybrid capacitors [104,105].

A 3D hierarchical graphene/PPy aerogel (GPA) was fabricated using GO and 1D PPy nanotubes [106]. The as-formed GPAs exhibited low density, large specific surface area, and high compressive strength, which are highly desired properties in porous materials. Moreover, the 1D nanotube geometry facilitated electron transport and allowed relatively larger surface areas than nanorods and nanowires. The aerogel contributed to the low density and formation of pore structures. The double-pore structure (between graphene–graphene sheets and in PPy) had a large surface area and exhibited outstanding electrochemical performance. Such 3D porous nanostructures have advantages in terms of the facile diffusion of electrolyte ions into and out of the inner region of the active materials and effective surface-area utilization [107]. Thus, it is expected that the 3D electrode structure of graphene-based hybrid capacitors will show a synergetic effect caused by materials hybridization and a unique hierarchical structure [108,109].

**Figure 16:**
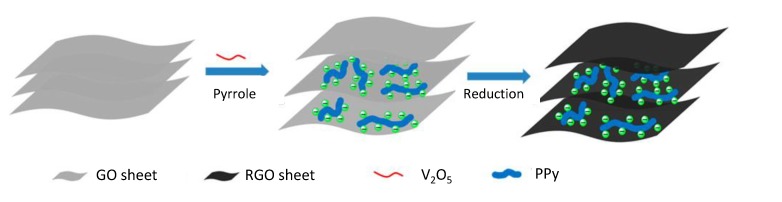
Scheme showing the synthetic route of PPy/RGO composites. With permission from [110]; Copyright 2014, American Chemical Society.

Another interesting example is a composite of PPy nanowires intercalated with RGO sheets (Figure 16) [110]. The intercalated PPy nanowires prevent restacking of the graphene sheets and allow the formation of an open 3D architecture for electrolytes. The combination of conducting polymers and graphene has led to rapid development of high capacitance for flexible electrochemical capacitors [111,112].

### 4.4. Summary

The electrochemical performance of pseudocapacitive conducting polymers and metal oxides can be improved through combinations with carbon materials. Table 3 summarizes the electrode materials for hybrid capacitors and their electrochemical performance. Vigorous on-going research and development are aimed at improving both the energy and power densities. However, the following issues should be further considered in the practical application of hybrid capacitors: the (i) different charge/discharge rates of EDLCs and pseudocapacitive materials and (ii) unstable charge/discharge cycling performance.

**Table 3:** Hybrid capacitor materials and electrochemical performance.

Materials ^(1)^	Electrode System ^(2,3)^	Electrolyte	Current Density or Scan Rate	Potential Range (V)	Specific capacitance (F·g^−1^)	Ref.
NiO/graphene	Two	1 M NaOH	7–20 A·g^−1^	1.5	130–440	13
Porous carbon/ Fe_2_O_3_ nanoparticles	Three	1 M Na_2_SO_3_	0.5–10 A·g^−1^	1.0	119–235	14
α-Fe_2_O_3_/Graphene	Three	1 M Na_2_SO_4_	3–10 A·g^−1^	1.0	98–306	15
Ni graphene aerogels	Two	6 M KOH	2–20 A·g^−1^	1.0	186–366	85
CoO carbon nanoflakes	Three	2 M KOH	10 mA·cm^−2^	0.75	476	86
Faradaic CNTs	Three	0.5 M H_2_SO_4_	3–100 mV·s^−1^	0.9	75–260	87
Nanoporous CuO/active carbon	Two	3 M KOH	1–10 A·g^−1^	1.4	54–72	88
Co(OH)_2_/graphene	Three	6 M KOH	2–10 A·g^−1^	0.7	356–532	89
PANI/RGO	Three	1 M H_2_SO_4_	0.45 A·g^−1^	0.8	431	90
PANI/CNF	Three	1 M H_2_SO_4_	0.3–10 A·g^−1^	1.2	400–557	91
PANI/N-doped CNTs	Three	0.1 M Na_2_SO_4_	50 mV·s^−1^	1.0	250	92
PPy/graphene	Two	1 M H_2_SO_4_	0.1 A·g^−1^	1.0	277	94
Ni–Mn–RGO	Two	1 M KOH	2–10 A·g^−1^	1.6	724–1985	95
3D carbon/CoNi_3_O_4_ asymmetric	Two	3 M KOH	1–100 mA·cm^−2^	1.8	42–64	96
*N*-doped carbon/PANI asymmetric	Two	1 M Na_2_SO_4_	0.5–20 A·g^−1^	1.1	75–113	97
WO_3_/PPy nanowire asymmetric	Three	3 M NaOH	0.7–7 mA·cm^−2^	0.6	250–800	98
Mn–Ni–Co oxide nanowire/RGO asymmetric	Three	6 M KOH	1–20 A·g^−1^	0.5	404–638	99
PEDOT/ROG/CNF asymmetric	Two	1 M H_2_SO_4_	0.1–2 A·g^−1^	1.0	50–60	100
MnO_2_/GO asymmetric	Two	1 M Na_2_SO_4_	0.1–2 A·g^−1^	2.0	41–84	101
RGO/MnO_2_ asymmetric	Three	1 M Na_2_SO_4_	0.1–1 A·g^−1^	1.5	217–243	102
CNF/GO/PANI	Two	1 M H_2_SO_4_	2 A·g^−1^	0.8	479	103
Ni/graphene/CNT	Two	6 M KOH	0.2–1.0 A·g^−1^	0.8	100–105	104
Graphene/PANI nanorods	Three	1 M H_2_SO_4_	1–8 A·g^−1^	0.7	836–1665	106
3D CoMoO_4_/graphene	Three	2 M KOH	1.5–85 A·g^−1^	0.9	1101–2741	108
Graphene/CNT/Mn	Two	2 M Li_2_SO_4_	1.9 A·g^−1^	1.6	1108	109
PPy nanowire/RGO	Two	PVA–H_2_SO_4_ gel	1–20 A·g^−1^	0.8	361–434	110
Porous graphene/PANI	Two	1 M H_2_SO_4_	1–8 A·g^−1^	0.8	458–864	111
Porous graphene/PANI	Two	1 M H_2_SO_4_	0.5–10 A·g^−1^	0.7	362–385	112

^(1)^ The term “asymmetric” is given when the capacitor cell has a cathode and an anode consisting of different materials, namely asymmetric electrode configuration. ^(2)^ The “Two” indicates capacitor cells composed of two electrodes (anode/cathode), separator, and electrolyte, containing symmetric or asymmetric electrode configurations. ^(3)^ The “Three” indicates electrode measuring systems composed of working electrode, counter electrode, and reference electrode in an electrolyte.

## 5. Outlook

Exploration of better electrode materials has been extensive over the few past decades. First, the surface area of the electrode/electrolyte interface must be increased in order to enhance the charge storage capacity in EDLCs. The use of a porous electrode with a higher specific surface area may increase the specific capacitance of an EDLC. Since ion desolvation occurs in pores smaller than the solvated ions, increased capacitance can be obtained from electrode materials with sub-nano-size pores. Meso- or macro-pores ensure the accessibility of ions. Thus, there have been many attempts to develop hierarchical nanoarchitectures with both micro- and macro-pores, in hope of fabricating better electrochemical capacitors. Although they are capable of providing high power for long cycles, EDLCs have a relatively low energy density. Therefore, future development is projected to still focus on the optimization of existing electrode materials and the creation of new materials for achieving energy densities approaching those of batteries.

Pseudocapacitors have significantly higher faradaic capacitance than double-layer capacitance. In general, conducting polymers and metal oxides render pseudocapacitance via a faradaic reaction. Enhancing the surface area of electrode materials is still an important factor in pseudocapacitors, as it is with EDLCs. In most cases, the faradaic reaction for pseudocapacitance occurs at the surface of the electrode materials. Nanostructured pseudocapacitive materials with increased surface area, therefore, have been developed and their morphology has been precisely tailored. Typically, fibrous and tubular materials exhibit higher capacitance than other morphologies. Combining different pseudocapacitive nanomaterials can result in excellent electrodes with improved electrochemical performance. However, the excellent performance of pseudocapacitors tends to degrade quickly upon cycling.

Various hybrid capacitors have been demonstrated as attractive alternatives to only EDLCs or only pseudocapacitors. The drawbacks of conventional EDLCs and pseudocapacitors, such as limited energy density and poor cycling stability, can be overcome by employing rational hybrid systems of pseudocapacitor (faradaic)-like and EDLC (nonfaradaic)-like electrodes, thereby producing a high working voltage and capacitance. There is growing demand for the development of electrochemical capacitors whose performance exceeds that of batteries. Nanomaterial-based hybrid capacitors have the potential to fulfill these requirements.

As a result, it is expected that future extensive research into nanomaterial-based electrochemical capacitors will offer great potential for the construction of next-generation energy storage devices. In particular, the development of nanomaterial-based wearable or flexible high-performance electrochemical capacitors is expected in the near future.

In this review, we have focused on nanostructured electrode materials. However, in addition to electrode materials, several factors such as electrolytes, separators, and binders also significantly influence the performance of electrochemical capacitors. First, different types of electrolytes, which are based on aqueous solutions, organic solvents, and ionic liquids, have been used in electrochemical capacitors. In general, aqueous gel electrolytes operate within a narrow potential range with a maximum of 1 V, leading to low cell voltage, which in turn limits energy and power densities. Moreover, water evaporation during the operation of these electrolytes over a wide temperature range negatively affects the performance and long-term stability of the devices. Ionic liquid gels, in contrast, are thermally stable, nonvolatile, nonflammable, and nontoxic over a wide potential range of 3.5 V. Sometimes, redox mediators can be introduced into the electrolyte to facilitate the redox reaction, thereby increasing the pseudocapacitance [113]. Likewise, electrochemical capacitors mostly use solution-phase electrolytes, which are prone to leaking. Recently, flexible, wearable electrochemical capacitors have been of particular interest as a mobile power supply for future flexible electronics. However, electrolyte leaking can be problematic in these flexible or wearable devices. All solid-state electrochemical capacitors, in which electrolyte gels function as the separator, can be an alternative to circumvent electrolyte leaking.

**Figure 17:**
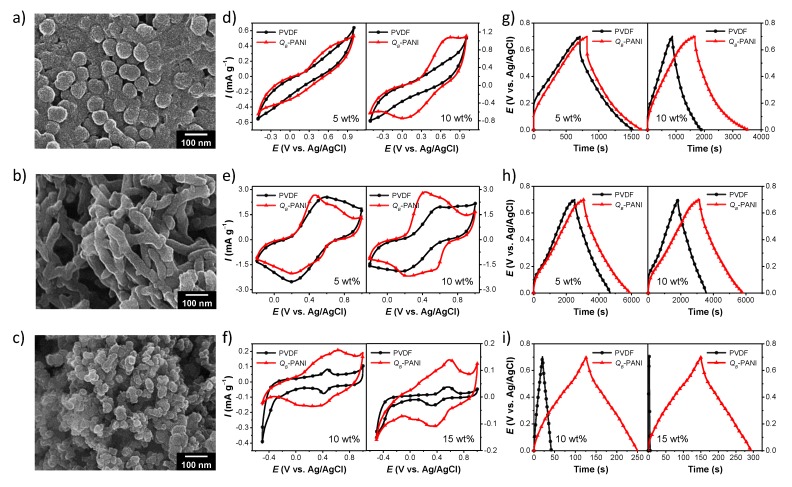

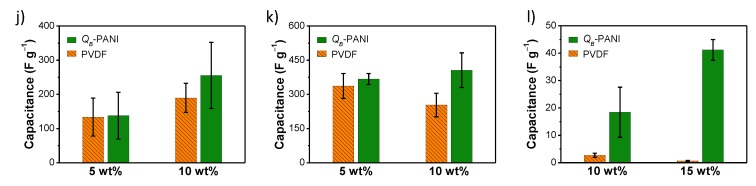
Electrode binder performance of an intrinsically conductive PANI glue: SEM images of nanoparticle electrodes prepared with a 10 wt.% PANI binder content: (**a**) PPy nanospheres, (**b**) PANI nanofibrils, and (**c**) carbon black. Representative CV curves and galvanostatic charge–discharge curves of the electrodes recorded at a scan rate of 25 mV·s^−1^ and a current density of 0.1 A·g^−1^, respectively, in 1 M sulfuric acid: (**d** and **g**) PPy nanospheres, (**e** and **h**) PANI nanofibrils (5 wt.% and 10 wt.% binder contents), and (**f** and **i**) carbon black electrodes with 10 wt.% and 15 wt.% binder. Gravimetric discharge capacitances calculated from the charge–discharge curves are presented in the histograms: (**j**) PPy nanospheres, (**k**) PANI nanofibrils, and (**l**) carbon black. With permission from [114]; Copyright 2014, The Royal Society of Chemistry.

Nonconductive polymers such as polyvinylidene fluoride and polytetrafluoroethylene are widely used as binders to construct electrodes. These polymers facilitate good adhesion between electrode materials or between electrode materials and current collectors. However, intrinsically, they are insulators and thus function as resistances that degrade the electrode performance. To circumvent this problem, conductive fillers have been introduced into the insulating polymers. However, it is difficult to achieve uniform dispersion of the fillers in the polymer matrix for obtaining desirable properties. Notably, an intrinsically conductive solution-processable PANI binder has very recently been prepared, in which PANI was treated with three organic additives as a ternary dopant [114]. The resulting PANI binders had abundant oxygenated functional groups such as sulfonate and hydroxyl groups, which facilitated ion transport. The polar groups of the additives provide adhesive properties via hydrogen-bonding or dipole–dipole interactions. The substituted alkyl chain may further afford adhesive properties to nonpolar surfaces through van der Waals interactions. Consequently, the PANI binders showed good conductivity and adhesive properties without the use of additional conductive fillers or heat treatment. Figure 17 shows the performance of a PANI binder in detail. PANI as a binder was found to significantly enhance the electrode performance of pseudocapacitive (PPy nanospheres and PANI nanofibrils) and EDLC (carbon black) nanomaterials. There is a still need for developing alternative efficient electrode binders capable of improving the device performance.

Additionally, the performance of electrochemical capacitors also depends on the microarrangement of the electrode materials in the cell. For example, capacitor applications are often constrained by volume rather than by weight. Unfortunately, nanomaterial-based electrochemical capacitors typically exhibit low volumetric capacitance. Therefore, increasing the packing density of nanomaterials inside the electrode is one of the critical issues in electrochemical capacitor research.

In summary, there are key challenges to achieving advances in electrochemical capacitor research:

(i) control of the 3D structure of electrode materials in the nanometer regime,
(ii) battery-like hybrid capacitors with both high energy and power densities,
(iii) flexible, all-solid-state devices,
(iv) use of intrinsically conductive binders or no binder,
(v) increased volumetric capacitance.

In conclusion, the electrochemical capacitor market continues to grow and expand into a greater number of applications. Continuous research on electrode materials, cell assembly, and entire cell systems should yield electrochemical capacitors that exhibit the excellent properties of both conventional capacitors and batteries and might ultimately facilitate the emergence of new types of energy storage devices.
